# Pedicled buccal fat pad flap for intraoral malignant defects: A series of 29 cases

**DOI:** 10.4103/0970-0358.53010

**Published:** 2009

**Authors:** Jayanta Chakrabarti, Rohit Tekriwal, Arun Ganguli, Saradindu Ghosh, Pranay K. Mishra

**Affiliations:** Department of Surgical Oncology, Cancer Centre, Welfare Home & Research Institute, Thakurpukur, Kolkata - 700063, West Bengal, India

**Keywords:** Buccal fat pad, Oral cancer, Pedicled flap

## Abstract

A buccal fat pad (BFP) as a flap for reconstruction of defects in the oral cavity has been described for a variety of benign conditions. We describe the indications, advantages, and complications of the BFP flap and report our clinical experience with the flap for intraoral reconstruction after tumor removal. From 2005 to 2008, we analyzed 29 patients in the age range of 32 to 82 years old who underwent a pedicled BFP flap reconstruction for oral defects after intraoral tumor removal. Postoperative wound healing and complications including any recurrence was followed-up prospectively. Most of the patients had an uneventful immediate postoperative period with signs of buccal fat pad epithelialization by the end of the first week and complete epithelialization at the end of the first month. On continued follow-up, a linear band of fibrous tissue under the epithelialized mucosa replaced the once reconstructed buccal fat pad. Three patients had varying degrees of hemorrhage: one of them had hematoma that healed with severe fibrosis and of the remaining two, one had a partial flap loss and one had a complete flap loss. Judicious use of buccal fat pad reconstruction offers a simple, convenient, and reliable way to reconstruct small to medium defects of the oral cavity with low morbidity, even in older patients who would not be able to tolerate time-consuming flap reconstruction procedures.

## INTRODUCTION

A pedicled buccal fat pad (BFP) flap was first described in 1977 by Egyedi[1] for the closure of oroantral communications after oncological resections. In 1983, Neder[2] utilized the buccal fat pad as a free graft in the oral cavity. In 1986, Tideman *et al.,*[3] showed that the pedicled buccal fat pad flap is epithelialized within 3 to 4 weeks and therefore cover with a skin graft is not required. Since then, there have been several studies on the use of this flap for closure of oroantral and oronasal communications secondary to exodontias. It was only after the onset of this century that Rapidis *et al.,*[4] Hao,[5] and Dean *et al.,*[6] used pedicled BFP flaps for reconstruction of medium sized post-surgical oral defects for malignant lesions. In 2005, Amin[7] showed how effectively a buccal fat pad can be utilized in post partial maxillectomy defects for neoplastic diseases. The procedural simplicity, with very low complication rates and excellent functional outcome, encouraged us to use a pedicled BFP as a reconstruction means for selective intraoral cancers.

### Anatomy

The buccal fat pad lies in the masticatory space between the buccinator muscle medially and the masseter muscle laterally, and it is wrapped within a thin fascial envelope. The BFP is divided into 3 lobes (anterior, intermediate, and posterior). The posterior lobe has four extensions (buccal, pterygoid, pterygopalatine, and temporal).

Several nutritional vessels exist in each lobe and together form a subcapsular plexus.[8] The principal arteries supplying the BFP are derived from the buccal and deep temporal branches of the maxillary artery, from the transverse facial branch of the superficial temporal artery and from a few branches of the facial artery.[3] Morphologically, the buccal fat pad is quite different from subcutaneous fat and is similar to orbital fat.[9]

The mean volume of BFP is 10 ml, with a mean thickness of 6 mm[10] and an approximate weight of 9.3 gm. It is capable of covering small to medium defects about 4 cms in diameter.

The physiological functions of BFP are 1) to fill the masticatory space, acting as cushion for the masticatory muscles, 2) to counteract negative pressure during suction in a newborn, and 3) as a rich venous net, with valve like structures, possibly involved in the exo-endocranial blood flow through the pterygoid plexus.[11]

## MATERIAL AND METHODS

This study included a series of 29 cases (21 males / 8 females) from 2005 to 2008, where buccal fat pad reconstruction was carried out after intraoral excision of squamous cell carcinomas [Table 1]. The age of patients ranged from 32-82 years.

**Table 1:** Patient characteristics, tumor description and postreconstruction outcome of patients in current series

*Age (yrs)*	*Site*	*Size (cms)*	*Type*	*Node*	*Imm post op*	*Healing*	*Fw up (months)*	*Rec.*
66	BM	3 × 2	VC	-	-	F scar	12	
56	BM	2.5 × 1.8	VC	-	-	P	10	
53	BM+AOM	5 × 3	Mic SCC	-	-	P	30	
60	BM+GB	4 × 3	VC	-	-	P	29	
54	BM+AOM	3.5 × 3.5	VC	-	-	P	5	BM leuko
82	BM	3.5 × 3	VC	-	Hm	Delayed	12	
60	Lip+BM	4 × 2	SCC	-	-	P	12	Upper lip
60	BM	4 × 3	MD SCC	-	-	P	3	Nodal
52	BM+AOM	4 × 3	Mic SCC	-	-	P	3	
56	BM+AOM	3 × 2.5	WD SCC	-	-	P	27	
60	BM	4 × 5	WD SCC	-	-	P	1
73	AOM	5 × 4	WD SCC	(0/5)	-	F scar	27	
65	BM	5 × 4	WD SCC	-	-	P	9	Nodal
52	GB	3.5 × 1.5	WD SCC	-	-	P	18	
67	RMT	3 × 2	VC	-	-	F scar	12	Leuko
53	BM	2.5 × 2	VC	-	-	P	15	BM leuko
65	BM	2.2 × 2	WD SCC	-	-	P	16	
57	AOM	1 × 0.5	VC	-	-	P	6	
50	BM	2.6 × 1.5	WD SCC	(0/10)	Hg	P. loss	7	
55	AOM+BM	3.5 × 3.5	MD SCC	(0/7)	-	P	6	
45	BM	2 × 1.5	WD SCC	(0/7)	-	P	6	
30	BM	3 × 1.7	Mic SCC	-	-	P	1	
60	AOM	4.5 × 3.2	WD SCC	-	-	OCF	5	
63	BM	5 × 3	WD SCC	-	-	P	22	
53	BM	2.8 × 1.8	WD SCC	-	-	P	12	
65	BM	2 × 2	WD SCC	(0/5)	-	P	12	Local
62	BM	4 × 3	WD SCC	(0/6)	-	P	6	
57	BM	3 × 2.5	WD SCC	-	Hg	C. loss	12	Nodal
42	BM	3 × 1	PD SCC	-	-	Leuko on BFP	22	BFP leuko

BFP – buccal fat pad; Imm Post op – immediate postoperative period; Fw up – follow up; BM – buccal mucosa; AOM – angle of mouth; GB – gingivobuccal; Lw – lower; Adj – adjacent; RMT – retromolar trigone; VC – verrucuous carcinoma; Mic – microinvasive; SCC – squamous cell carcinoma; MD – moderately differentiated; WD – well differentiated; PD – poorly differentiated; Hm – hematoma; Hg – hemorrhage; F scar – fibrous; P – proper healing; OCF – orocutaneous fistula; C. loss – complete loss; leuko – leukoplakia; rec – recurrence

Lesions situated on the buccal mucosa, angle of mouth, and retromolar trigone with well defined margins were selected to be suitable for buccal fat pad reconstruction because of the proximity of the buccal fat pad. Patients with a history of prior radiotherapy to the site or neo-adjuvant chemotherapy were excluded because of the uncertainty of the size of the primary on presentation.

Due to the limitation of the size of the buccal fat pad to effectively cover the excised area, defects more than 5cms, or where the underlying bone (upper and lower alveolus, ascending ramus of mandible) has been exposed were not reconstructed with the buccal fat pad.

Patients found to have accompanying submucous fibrosis and with very poor oral hygiene and unhealthy mucosa surrounding the lesion were not included for buccal fat pad reconstruction.

The mean maximum dimension of the defect was 3.365cms with a range of 1-5 cms (median – 3.5 cms, standard deviation 0.988).

Out of 29 patients who underwent operation, 4 had histologically positive margins and opted for adjuvant radiotherapy instead of re-excision.

Since a majority were early cases with nonpalpable neck nodes (N_0_), the neck was not intervened in these cases whereas patients with single palpable neck node (all in Level Ib and less than 1.5 cms) (N_1_) underwent a supraomohyoid neck dissection (SOHND). There were no cases with multiple neck nodes i.e., N_2_ onwards.

**Figure 1:**
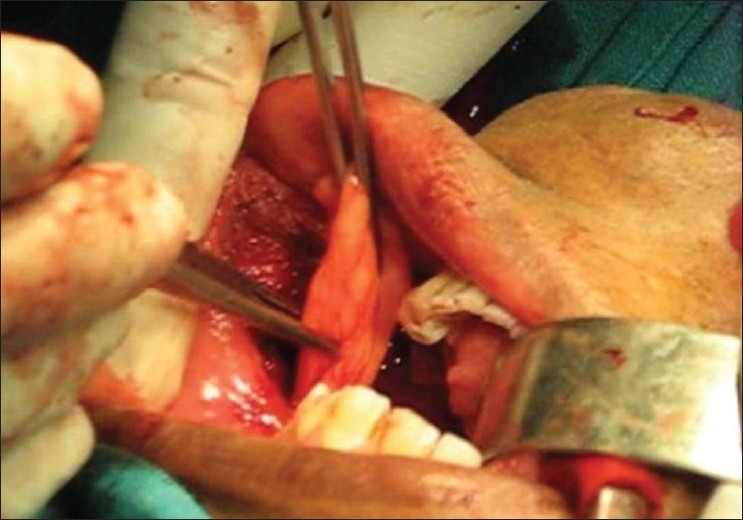
Mobilization of buccal fat pad flap

**Figure 2:**
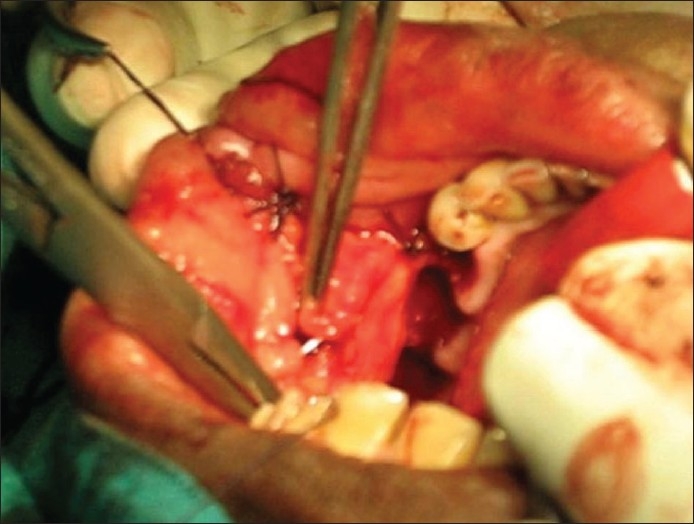
Suturing of buccal fat pad flap to cover the defect

Postexcision, the buccal fat pad was gently delivered atraumatically using nontoothed/deBakey's forceps (avoiding sharp/crushing instruments) [Figure 1] from the masticatory space by blunt dissection with hemostasis (using bipolar coagulation), keeping the fascial envelope intact so as to preserve its vascular supply and cover the defect [Figure 2]. It has been observed that all the defects could be adequately covered with the buccal fat pad without breaching the fat envelope or stretching it too thin. The buccal fat pad was sutured to the defect margins by simple, interrupted sutures with 3-0 Polyglactin 910 (vicryl^®^). Use of suction during the delivery of buccal fat was strictly avoided.

**Figure 3:**
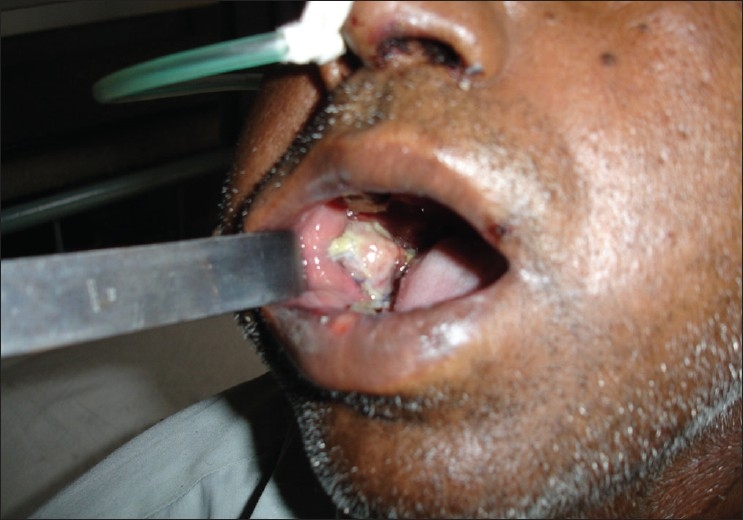
BFP on 5^th^ postoperative day showing minimal bulge in the initial period

The buccal fat pad was assessed daily and oral rinsing was probihited for the first 4-5 days. The patient was put on nasogastric feeding for 4-5 days [Figure 3]. The surgical field was cleaned twice daily with saline soaked gauze.

**Figure 4:**
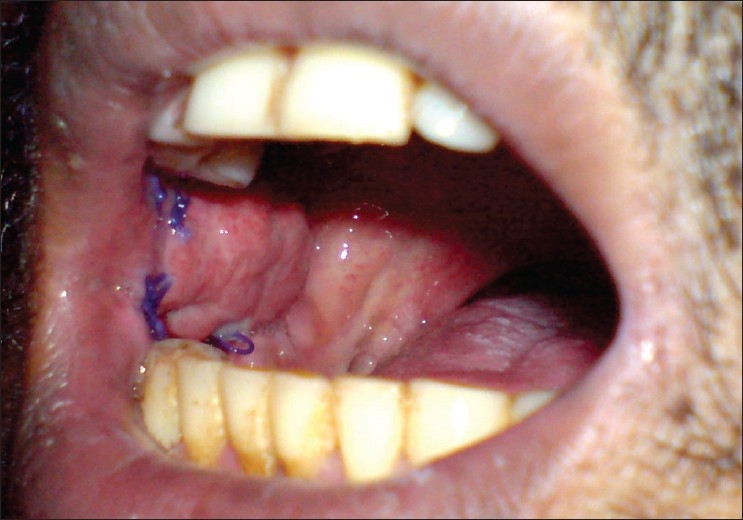
BFP at 1 month with epithelialization almost completed

**Figure 5:**
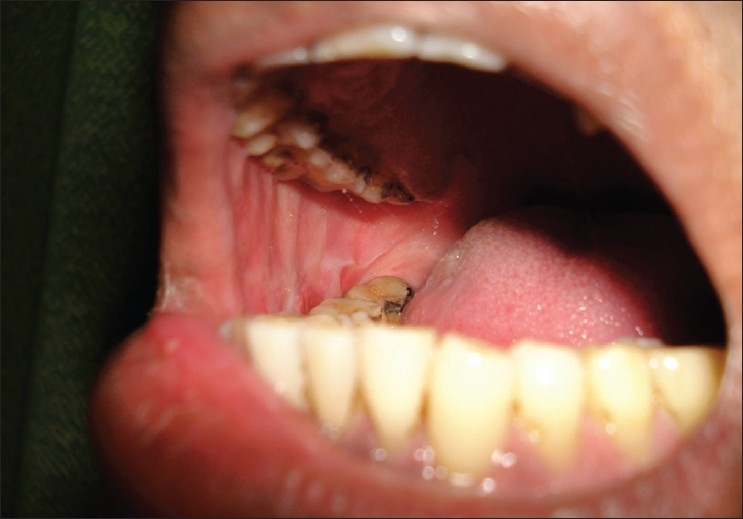
BFP at 3 months showing normal healing with adequate mouth opening

The patients were evaluated on the 7^th^ day. They were then evaluated after 2 weeks, 1 month [Figure 4], and at every 3 months for the first 2 years [Figure 5].

## RESULTS

In the immediate postoperative period, most of the patients fared well except for 3 patients. One of them had a hematoma in the buccal fat pad, which healed with severe fibrosis later on. The remaining 2 patients had a hemorrhage from the operative site leading to a complete loss of the BFP in one patient and a partial loss in the other, which healed with moderate fibrosis. In one case, the hemorrhage was severe enough to mandate exploration. Although no definite named vessel could be identified, it could be attributed to any of the vascular pedicles of the buccal fat pad. The second case was managed with local application of topical hemocoagulase, surgicel and gentle manual compression to preserve the integrity of the flap.

In all the patients who had an uneventful immediate postoperative period, signs of buccal fat pad epithelialization had started by the end of the first week and the BFP was completely epithelialized at the end of the first month [Figure 4].

Three months later, at the second follow-up visit [Figure 5], most of the patients had the buccal fat pad replaced by a thin whitish streak covered by normal mucosa, with very minimal fibrosis.

We lost 3 patients to follow-up and the remaining 26 patients have a median follow-up of 12.33 months with a minimum follow-up time of 1 month and a maximum follow-up of 30 months.

Two patients had nodal recurrence (both Level II, one N_1_, and one N_2_) on 3 and 9 months of follow-up, respectively (N_0_ on presentation, therefore observed) and were treated with a modified radical neck dissection followed by radiotherapy. Local areas in both the cases were free of recurrences.

One patient had a local recurrence after 12 months of follow-up and was subjected to revisional surgery and adjuvant radiotherapy.

One patient developed a lesion on the upper lip on the same side close to the previous lesion, at 12 months follow-up (probably a new primary) and was treated by surgical excision with adjuvant radiotherapy.

On follow-up, 1 patient presented with leukoplakia on adjacent mucosa, 2 patients had leukoplakia on the opposite buccal mucosa, which were proven histologically and managed conservatively. Another patient had a leukoplakic patch on the site of buccal fat pad reconstruction itself and is under close follow-up.

**Figure 6:**
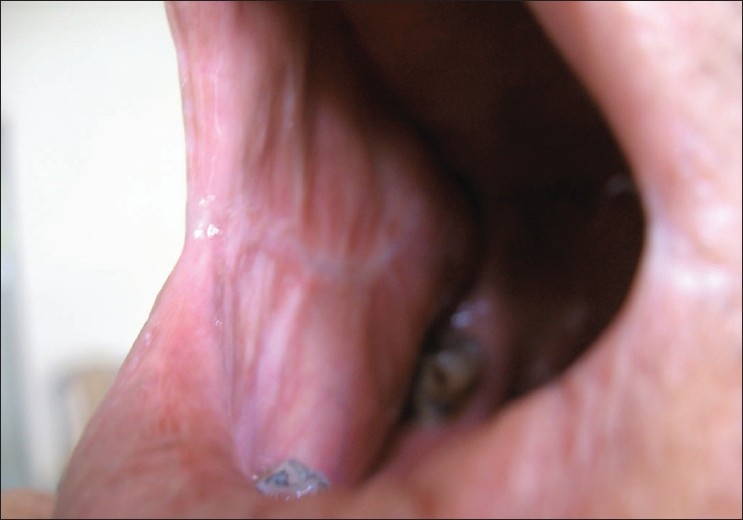
BFP at 18 months - normal mucosa almost replacing the buccal fat pad flap

It is important to mention that all the patients with normal follow-up have a linear band of fibrous tissue with overlying mucosa replacing the once reconstructed buccal fat pad. Although no biopsies from completely healed sites were taken in our study, during local examination of the healed sites, a thin fibrous band under normal mucosa could be felt [Figure 6].

The mouth opening was satisfactory (three fingers) in all the patients, including those who received adjuvant radiotherapy.

## DISCUSSION

The buccal fat pad remains constant in terms of size even in extreme weight gain or losses, and it is not proportional to total body fat.[3,12]

The buccal fat pad becomes an ideal choice for medium to even larger intraoral defects, because local flaps such as the buccal advancement flap, palatal pedicled flap, double layered closure flaps using buccal and palatal tissues, and other such procedures disappointingly produce large denuded areas and are unsuitable for large defects.[13,14] Distant flaps (Temporalis muscle or Nasolabial flaps) although used for intraoral reconstruction are generally not used because of their invasiveness.

The buccal fat pad has been used to reconstruct defects in the hard palate, the soft palate (up to midline), the retromolar trigone, the buccal mucosa, the anterior tonsillar pillar, the superior alveolar ridge (up to the canine region), and the superior buccal sulcus.[15] It can be used alone or in combination with other flaps such as the pedicled temporalis muscle myocutaneous flap (Samman *et al*.[16]) or the pectoralis major myocutaneous flap,[6] where the posterior portion of the defects (palatal region and tonsillar pillar) were reconstructed by the buccal fat pad, leaving the anterior and inferior portion to be covered by myocutaneous flaps.

In our series, a few cases with lesions involving the angle of mouth had required additional flaps for lip reconstruction.

The pedicled buccal fat pad flap has also been used to close oroantral and oronasal fistula[15,16] and to cover the nasal or antral surface of bone grafts used for maxillary reconstruction.

The body and buccal extension (accounts for almost half of the total volume of buccal fat pad) are accessible through the oral cavity. In all of our cases, the buccal fat pad flap could be easily extracted through the resection bed (technique described earlier).

The other approach for buccal fat pad access is through an incision along the superior vestibular sulcus at about the level of the upper second molar and cutting backwards about 2 cms. The incision cuts the mucosa and the buccinator exposing the maxillary periosteum and the buccal fat pad.[6] The choice of approach depends upon the defects to be closed. The above metioned approach is suitably applicable to defects in the post alveolus or upper gingivobuccal sulcus and also for partial maxillectomy defects.

Various authors previously have recommended optimum defects to be reconstructed by the buccal fat pad. After an overall review of their recommendations, a guideline may be adopted, which suggests a) maxillary defects of approximately 4 cms and b) buccal or retromolar defects up to 6 cms can be covered with the buccal fat pad.[3]

A fact has to be borne in mind that excessive stretching in the flap invariably impairs the vascularity, so closure of larger defects cannot be guaranteed without producing flap necrosis or creating new fistula.[18] Taking this into consideration, the maximum size of a defect reconstructed using the buccal fat pad was 5 × 4 cms only. The lesions in our series were T1 or early T2 and excisions were carried out with at least 1 to 1.5 cm margins at the surface and including the underlying buccinator muscle to ensure an adequate three-dimensional excision. Mucosal margins from four areas were separately sent for histological examination. Four patients had only focal positive margins at single sites. This finding can probably be attributed to field cancerization changes commonly associated with oral cancers and is impossible to detect peroperatively.

The buccal fat pad is epithelialized in a few weeks, and thus obscures any need for additional skin grafts.[3] In our cases, we observed the epithelialization of the buccal fat pad pedicle after the second week with complete epithelialization in 4 to 6 weeks. Sometimes, the buccal fat pad protrudes exclusively into the oral cavity [Figure 3] but flattens progressively[18,19] without affecting the epithelialization process.

The histological nature of the healing process of the buccal fat pad was first reported by Samman *et al*.[16] He observed that no fat cells were seen in sections taken from healed sites, indicating fibrosis of the fat tissue and the reconstructed area was covered by parakeratotic stratified squamous epithelium.

Recovery of sensation in the reconstructed area takes about 4 weeks.[20]

Commonly reported complications with buccal fat pad reconstruction are hematoma, partial necrosis, excessive scarring, and infection.[4,14] Severe arterial bleeding like in one of our cases has also been reported before.[21]

Radiotherapy, if necessary, is best begun once epithelialization is complete, but does not seem to influence the success of the reconstruction.[3,16]

Four of our patients received radiotherapy; two of them have a follow-up of 12 months and 29 months with perfectly healed sites of reconstruction. The radiation-induced fibrosis was minimal because complete epithelialization over a well-vascularized BFP flap occurred prior to radiotherapy, although precautionary measures (graduated metallic mouth opener and regular exercise) to prevent postradiation trismus were undertaken in these cases. Out of the remaining two patients, one patient was lost to follow-up immediately after radiotherapy and the other developed Level 1b nodal recurrence at 6 months and was treated with definitive surgery.

The success rate of the buccal fat pad in the reconstruction of oral defects is quite high in all of the previous studies and is similar in our series as well. Its main advantages are that a) it is a quick, simple, and easy flap to use, b) heals with minimal scarring, c) has a very low morbidity, d) failure rate is very low, and e) can be used along with other flaps. The drawbacks of the buccal fat pad flap are a) it can only cover small to medium defects and b) because of its thinness, it cannot provide any bulk.

Contrary to the western world, where patients are diagnosed at an early stage, the majority of our patients present at an advanced stage. So inspite of having a high incidence of oral cancers, many of them are not suitable candidates for buccal fat pad reconstruction.

The subset of our patients who underwent buccal fat pad reconstruction had remarkable functional outcomes, and the follow-up trend shows equivalent oncological outcomes.

In conclusion, judicious use of buccal fat pad reconstruction offers an easy way to reconstruct small to medium defects of the oral cavity with low morbidity.
